# Beyond spherical vesicles: morphological diversity of extracellular vesicles in health and disease

**DOI:** 10.1080/07853890.2026.2663575

**Published:** 2026-05-04

**Authors:** Manuel Adrián Velázquez-Cervantes, Nora Alma Fierro, Ricardo Lozano-Hernández, Julio César Carrero

**Affiliations:** ^a^ Immunology Department, Instituto de Investigaciones Biomédicas, Universidad Nacional Autónoma de México, México City, México; ^b^ Lab Medical Center, Unidad Andrológica y de Reproducción, San Cristóbal, Edo Táchira, Venezuela

**Keywords:** Extracellular vesicles (EVs), morphology variation, EV biogenesis, Cryo-TEM, infectious and non-infectious diseases

## Abstract

**Background:**

The field of extracellular vesicles (EVs) has advanced considerably in recent years with new findings stemming from technical improvements in their isolation and characterization, expanding their potential as tools in vaccines, drug delivery, and timely diagnosis using biomarkers. Consequently, a new area of interest has recently gained prominence: the study of changes in EV morphology and their potential involvement in diseases.

**Discussion:**

Using cryo-TEM analysis, a technique that helps preserve the near-natural state of EVs, multiple morphological and structural variants have been demonstrated in isolates from bodily fluids, cell cultures, and cell lines of mammals, as well as some protozoa and plants. In addition to the classic sphere with a lipid bilayer and hyaline content, pleomorphic, tubular and sac-like EVs, with double, multilayered or lamellar membranes, and in some cases, with electrodense content, have been described. Furthermore, several studies have found alterations in the morphological pattern and/or proportions of these morphological variants in diseases associated with cellular or metabolic dysfunction such as Parkinson’s and diabetes, as well as in infectious diseases such as Zika virus and prions. Here, we review the key findings that have propelled the field, provide a catalog of the EVs variants identified to date, discuss mechanisms underlying their formation and their potential biological implication in the course of various pathologies, and identify key challenges that need to be addressed.

**Conclusion:**

The information analyzed demonstrate that EVs are highly variable structures, not only in their content but also in their morphology, and that this variation could be related to cellular, metabolic, and infectious pathologies. This underscore the need to understand the origin, regulation, and function of each morphological type of EVs, which could lead to their possible use as diagnostic or therapeutic tools in the future.

## KEY MESSAGES

1. Findings from the last decade using cryo-TEM in bodily fluids demonstrate the existence of morphological and structural variants of extracellular vesicles (EVs).
2. Emerging evidence from microorganisms and plants suggests that this phenomenon may represent a widespread biological process across nature.
3. Changes in EV morphological variants and their proportions have been reported in several diseases involving metabolic and cellular dysfunction, as well as in some infectious diseases.

## 1. Extracellular vesicles and their components

In the cellular dynamics, there are agents responsible for maintaining homeostasis by transporting proteins, lipids, metabolites, and nucleic acids, and by facilitating communication between cells. These structures, released by all living cells, are known as extracellular vesicles (EVs), spherical components whose main characteristic is that they are formed by a lipid bilayer [1–4]. The first reports on EVs date back to the 1940s, when Chargaff and West observed small products derived from the degradation of blood corpuscles with coagulation functions [5]. Later, in 1967, thanks to electron microscopy and biochemical analysis, it was observed that these products were vesicular in shape and contained biomolecules such as lipids, metabolites, and proteins. Additionally, it was determined that they originated from multivesicular bodies within cells [6,7]. Studies on glioma cell lines showed that vesicles were released through a process of exfoliation, and they were proposed to be called *‘*exosomes*’* [8]. Subsequently, in 1983, Johnstone identified that reticulocytes released vesicles loaded with transferrin receptors that were no longer needed by the cell, so they were initially thought to be *‘*bags of cellular waste*’* [9]. However, it was not until 1996 and 1998 that these vesicles were shown to have biological functions. When isolated from immune cells, they were found to have the ability to activate the immune response by promoting the maturation of antigen-presenting cells [10,11].

Currently, although there is no universal consensus on their definitive designation and classification, EVs are usually classified according to their cellular origin into at least two big groups: those derived from the plasma membrane (microvesicles or ectosomes) and those of endosomal origin (exosomes), each with well-defined characteristics [3,4,12–15]. Microvesicles or ectosomes range from 100 to 1000 nm in size and are formed by the outward budding from the plasma membrane. Annexin A1 and ARF6 have been reported as markers that define them [16–18]. Exosomes, on the other hand, range from 50 to 180 nm and originate from endosomes that form multivesicular bodies (MVBs) through inward budding. They are characterized by specific markers such as CD9, CD63, CD81, and Alix [16,18,19]. Additional EVs types have been reported, including apoptotic bodies [20], secretory autophagosomes/amphisomes [21], and migrasomes [22], among others.

**Figure 1.**
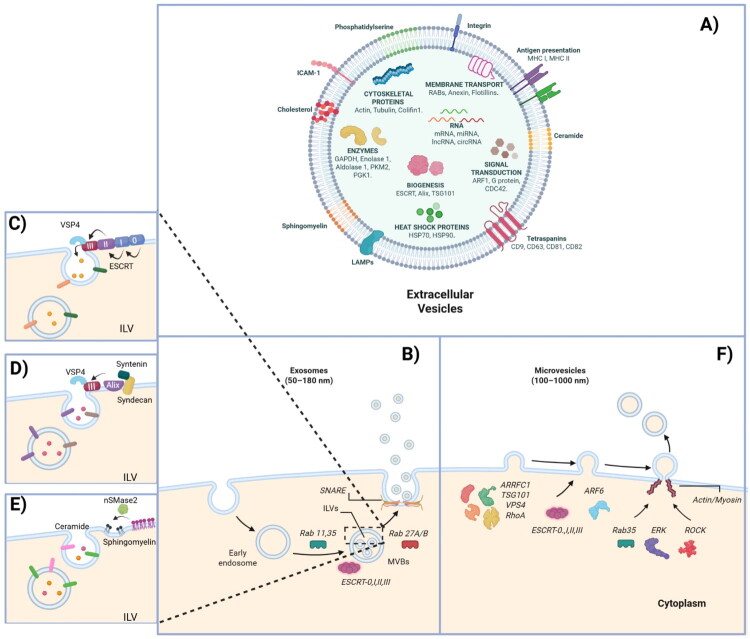
Extracellular vesicles content and biogenesis. (A) The main components of an EV are shown, including the bilayer structure composed of various lipids and proteins such as integrins, LAMPs, MHC-I, MHC-II, and tetraspanins, and the internal cargo consisting of heat shock proteins, cytoskeletal proteins, EV biogenesis proteins, signal transducers, membrane transporters, enzymes, and nucleic acids. (B) Biogenesis of exosomes, which originate from the endosomal pathway, forming an early endosome. The ESCRT complex is recruited to form ILVs that carry biomolecules depending on the cellular context in the MVB. These are then transported to the plasma membrane and interact with the SNARE complex to release the exosomes. (C) ESCRT pathway. (D) ESCRT-dependent pathway regulated by Alix with syntenin and syndecan. (E) ESCRT-independent pathway regulated by ceramides. (F) Biogenesis of microvesicles; elements such as ARF6, RhoA, TSG101, and the ESCRT complex are recruited to induce budding of the plasma membrane. Subsequently, ERK and ROCK are recruited to regulate actin and myosin for microvesicle fission. Created using BioRender.com.

The biomolecules contained in EVs depend on the cell type and cellular context conditions, but certain proteins, nucleic acids, and lipids are consistently present in these structures and have therefore been used as EV markers (Figure 1A). An example is the proteins ALIX and TSG101, which participate in protein cargo loading into the vesicles, and the heat shock proteins HSP70 and HSP90, which are part of the stress response and play an important role in antigen binding and presentation [23–26]. It has also been reported that the RAB, annexin and flotillin families are found within EVs and play an important role in their biogenesis, trafficking and fusion [23,24]. In addition, EVs of endosomal origin (exosomes) are enriched in a group of tetraspanins (CD9, CD63, CD81, and CD82) involved in cell fusion processes [15]. Within the EV cargo, many typical components have been described, including metabolic enzymes (GAPDH, enolase 1, aldolase 1, PKM2, PGK1, PDIA3, GSTP1, DPP4, AHCY, TPL1, peroxiredoxins, P4HB, LDH, cyclophilin A, FASN, MDH1, and CNP), ribosomal proteins (RPS3), transmembrane proteins (PIGR, LAMP1, and CD59), proteins involved in signal transduction (G proteins, ARF1, CDC42), cytoskeletal proteins (actins, tubulins, cofilin 1, ezrin, profilin 1, moesin, radixin, myosin, perlecan, THBS1, IQGAP1, keratins, gelsolin, fibronectin 1, and LGALS3BP), antigen presenting molecules MCH I, MCHII, and ubiquitin B and C. These studies of EV cargo have demonstrated the importance of these vesicles as transporters of biomolecules [3,23,24,27]. Interestingly, messenger RNA, non-coding RNA, and microRNA have also been found in EVs. The most commonly reported microRNAs are miR-1, miR-29a, miR-126, miR-214, and miR-320, which participate in angiogenesis, hematopoiesis, and exocytosis, and have been observed to play a role in cell-to-cell communication [27,28]. On the other hand, EVs, as mentioned above, are formed by a lipid bilayer consisting largely of phosphatidylserine, phosphatidic acid, cholesterol, ceramides, sphingomyelin, arachidonic acid, prostaglandins, and leukotrienes, which are key to the stability and structural rigidity of these structures (Figure 1A) [26].

The classic morphology of EVs is that of a spherical structure delimited by a lipid bilayer with a bright, low-electron-dense luminal content. This view has been challenged in recent years with the increasing description of morphological variants identified in bodily fluids of healthy individuals, with changes in their proportions in patients with certain diseases. Although the molecular mechanisms involved in their formation have not yet been demonstrated, EV morphology likely reflects both their biogenesis pathways and molecular composition. EVs of endosomal origin arise *via* ESCRT-dependent or independent mechanisms within multivesicular bodies, whereas plasma membrane-derived EVs are formed by outward budding coupled to cytoskeletal remodeling (see below). These distinct processes impose structural constraints that can influence vesicle size and curvature. In addition, EV morphology may be shaped by cargo loading and protein and lipid membrane composition, particularly factors affecting membrane rigidity and bending. Cellular conditions such as stress or activation state further modulate these processes, suggesting that EV structural heterogeneity could be functionally linked to their biological roles. In this review, we analyze the topic of morphological variation of EVs in health and disease in light of the most recent findings, summarized the number of variants identified, and speculate about the possible mechanisms of formation and their biological relevance.

## 2. Biogenesis of EVs

As mentioned above, EVs are released by all living cells. The biogenesis of these structures has been extensively characterized in mammalian cells but not in other eukaryotes and in prokaryotes. However, homologous components of the endosomal sorting complexes required for transport (ESCRT) machinery, essential for EV formation, have been identified in plants and fungi, and notably, proteins functionally similar to ESCRT-0 are present in early endosomes, while the ESCRT-I, ESCRT-II, and ESCRT-III complexes are conserved in MVBs [29–32]. On the other hand, EV biogenesis in bacteria is completely different. Gram-negative bacteria produce outer-inner membrane vesicles as part of the cell wall renewal process, as well as in response to physical, saline, or antibiotic-induced stress, and these vesicles may contain cell wall components, lipopolysaccharides, enzymes, and nucleic acids. In contrast, in Gram-positive bacteria, it has been proposed that vesicles originate from the cytoplasm through a normal physiological process [30,33].

### 2.1. Biogenesis of exosomes

As mentioned above, different types of EVs differ in their biogenesis. Exosomes have an endosomal origin, a process that begins with the formation of early endosomes, which subsequently recruit the endosomal sorting complexes required for transport (ESCRT) to generate late endosomes (Figure 1B). Within these, intraluminal vesicles (ILVs) are formed, giving rise to MVBs that eventually fuse with the plasma membrane to release exosomes [34]. The ESCRT complex, consisting of four protein complexes: ESCRT-0, -I, -II, and -III, plays a crucial role in this process. It is recruited to the cytosolic side of the endosomal membrane, a process that requires ubiquitination of the cytosolic tail of endocytosed receptors [35,36]. Subsequently, TGS101 protein associates with ESCRT-I, forming a complex that recruits ubiquitin and activates ESCRT-II. ESCRT-II then undergoes oligomerization and facilitates the formation of ESCRT-III, which plays a key role in sequestering MVB proteins and recruiting deubiquitinating enzymes that remove ubiquitin from the proteins incorporated into ILVs, forming part of the exosomal cargo. Finally, ESCRT-III is dismantled by an ATPase, allowing the complex to be recycled for other biological functions, such as targeting membrane proteins for lysosomal degradation [37].

In addition to ESCRT proteins, the RAB family of small GTPases regulates the intracellular trafficking of MVBs, including budding, vesicle and organelle mobility through cytoskeletal interactions, and docking of vesicles at the plasma membrane [38]. Specifically, RAB11 and RAB35 are associated with early endosome recycling and sorting, whereas RAB27A and RAB27B are related to late endosomes and secretory compartments involved in exosome release [39–41]. For exosome secretion into the extracellular space, the soluble N-ethylmaleimide-sensitive factor attachment protein receptor (SNARE) complex mediates the fusion of MVB and plasma membrane lipid bilayers. SNARE is composed of syntaxin, VAMP, and SNAP-25. The mechanism involves interaction between vesicular SNAREs (v-SNAREs) and target membrane SNAREs (t-SNAREs), leading to vesicle docking and membrane fusion, which allows exosome release (Figure 1B and C) [42–46] This process represents the ESCRT-dependent pathway of exosome biogenesis. On the other hand, exosomes contain phosphatidylserine, cholesterol, ceramides, sphingomyelin, LBPA, integrins, ICAM, MHC-I, MHC-II, CD9, CD63, CD81, CD151, TSPAN6, and TSPAN8. Proteins such as ALIX, TSG101, VPS54, HSP70, HSP90, syntenin, ubiquitin, RABs, GAPDH, PGK1, clathrin, G protein, and a range of different nucleic acids have commonly been found inside exosomes [47].

Other non-canonical ESCRT-dependent pathways have also been described, such as the Alix pathway, in which Alix interacts with syntenin and syndecan to recruit ESCRT-III and VPS4 for ILV formation (Figure 1D) [48,49]. On the other side, among the ESCRT-independent mechanisms, the ceramide pathway has been described. Ceramide, a cone-shaped lipid, plays a key role in ILV invagination within late endosomes. Neutral sphingomyelinase 2 (nSMase2) hydrolyzes sphingomyelin into ceramide, generating negative curvature in the endosomal membrane, which promotes inward budding and regulates the lipid and protein composition of ILVs [49]. Inhibition of nSMase2 leads to vesicle accumulation within MVBs and a consequent decrease in exosome secretion (Figure 1E) [50,51]. These pathways vary depending on cell type and external conditions affecting cellular homeostasis, which in turn influence EV secretion.

### 2.2. Biogenesis of microvesicles

Microvesicles originate from outward budding of the plasma membrane, using components similar to those involved in exosome formation, such as the ESCRT complex, which interacts with the arrestin-like domain-containing protein 1 (ARRDC1). ARRDC1, together with TSG101 and VPS4, promotes plasma membrane budding [16,52]. Before microvesicle release, actin filament reorganization is essential to generate the mechanical force required for vesicle budding. In this context, it has been described that GTPases such as ARF6, RhoA, and Rab35 regulate actomyosin contraction at the neck of emerging microvesicles. Specifically, ARF6 promotes vesicle detachment by activating phospholipase D, which recruits extracellular signal-regulated kinase (ERK). ERK then phosphorylates the myosin light chain, enabling actin-myosin contraction and microvesicle fission [53,54]. Moreover, Rho-associated protein kinase (ROCK) contributes to microvesicle release by activating LIM kinase, which phosphorylates cofilin, promoting actin filament extension and facilitating microvesicle detachment (Figure 1F) [55–57]. Microvesicles have been reported to be composed of membrane lipids such as phosphatidylethanolamine, phosphatidylcholine, sphingomyelin, phosphatidylserine, glycerophosphoserine, integrins, fibronectin, annexins, LFA1, CD9, CD14, CD81, CD82, CD105, EGFR, MDRP, and ICAM-1. Inside, RAB proteins, GTPases, TGS101, ROCK, HSP70, HSP90, actin, tubulin, ARF6, GAPDH, and various types of RNA can be found [47,58,59].

## 3. Microscopy of EVs

Techniques employed in the study of EVs include Western blotting, PCR, proteomics, nanoparticle tracking analysis (NTA), and electron microscopy. Over the past decade, electron microscopy has become one of the most widely used tools for morphological and structural characterization of EVs [15]. This technique encompasses modalities such as transmission electron microscopy (TEM), scanning electron microscopy (SEM), and cryo-electron microscopy (cryo-TEM) (Table 1).

**Table 1.** Comparison of types of electron microscopy including advantages and disadvantages.

Electron microscopy	Preparation	Resolution	Sample type	Advantages	Disadvantages
TEM	Fixation, negative staining, dehydration, chemical compounds	1,000,000x	Cellular and subcellular structures	High resolution Evaluation of shape heterogeneity and aggregation	Complicated sample preparation procedure Variable results No morphological details Artifacts (flattening, size reduction, salt crystals, protein aggregates) Long sample preparation time
SEM	Fixation, dehydration, high vacuum	100,00x	Cellular and subcellular structures	Three-dimensional images Obtaining images of bulky samples Depth of field	Low resolution at nanometer scale Complicated sample preparation procedure Long sample preparation time
Cryo-TEM	Vitrification	1,000,000x	Vesicles and biomolecules	No fixatives, chemicals, or dehydration Observe the sample in its near native state, without dehydration Minimal sample quantities can be analyzed Sample preparation in seconds Internal structures of the sample can be observed If a tomograph is connected, structures can be analyzed in 3D (Cryo-ET)	Structures with a thickness of 500 nm are not observed. Poor contrast. Crystalline ice contamination may occur. Low temperatures.

The development of electron microscopy dates back to the twentieth century, beginning with the invention of the TEM by Ruska and Knoll in 1932, who employed an electron beam to obtain high-resolution images [60]. Subsequently, in 1938, von Ardenne incorporated a scanning coil into the TEM design, leading Zworykin, Hillier, and Snyder to develop the SEM in 1942, which used secondary electrons to generate topographic contrast. Later, in the early 1980s, Dubochet introduced vitrification, solidifying a thin aqueous layer by rapid cooling within milliseconds, marking the advent of cryo-TEM. The low-temperature approach (−170 °C) of cryo-TEM minimizes electron-induced damage, preserving samples in their near-native state for extended periods [61,62].

### 3.1. Transmission electron microscopy (TEM)

TEM was the first electron microscopy technique developed to achieve high-resolution imaging of microscopic structures. Its operation relies on an electron beam, which provides resolutions between 0.2 nm and 2 nm. Sample preparation typically involves freeze-fracturing, negative staining, or vitrification [63]. The TEM electron beam operates at 80–300 kV and is generated from thermionic sources, such as tungsten filaments or lanthanum hexaboride (LaB_6_) crystals, or from field emission sources composed of fine lanthanum needles. LaB_6_ sources provide a higher current density and greater beam stability. The anode, operating at 40–400 kV, accelerates the electron beam, which is subsequently focused onto the sample by a system of electrostatic and electromagnetic lenses. The transmitted electrons are then magnified by objective lenses to produce the final image. The TEM can achieve magnifications up to 1.5 × 10^6^, enabling structural visualization at near-atomic resolution [63–66]

### 3.2. Scanning electron microscopy (SEM)

SEM is primarily used to examine the surface morphology, chemical composition, crystal structure, and size of samples such as EVs. Sample preparation typically involves chemical fixation, dehydration, and exposure to high vacuum conditions [67]. SEM can achieve magnifications up to 1 × 10^6^, allowing observation at the nanometer scale.

The electron beam operates under high vacuum (10^−6 ^Torr), minimizing collisions with residual gas molecules and enabling low-energy electron emission [68]. The technique employs beam energies between 2 keV and 40 keV, depending on the sample characteristics, and electron sources can include tungsten, LaB_6_, or field emission guns. Electromagnetic lenses focus the beam, which scans the sample surface to produce secondary electron signals. These signals are detected, transformed into images, and can be coupled with X-ray detectors for compositional analysis. SEM provides detailed information on microstructural features, such as fractures, particles, or mineral phases, as well as insights into the chemical, physical, and biological processes affecting the sample [68,69].

### 3.3. Cryogenic transmission electron microscopy (cryo-TEM)

Cryo-TEM allows ultrastructural details to be observed by freezing samples at −170 °C using liquid nitrogen or helium. Cryofixation is extremely important in this technique, a procedure based on ultra-rapid freezing (vitrification) of the sample allowing ice crystals to remain smaller than 10 nm and consequently avoids damage to the structures of the sample being analyzed. This is achieved by lowering the temperature of the sample below −180 °C at cooling rates of up to 106 °C/s. This vitrification process preserves the near-native structure of biological samples without the addition of any heavy metals or fixatives, which might cause artifacts, thereby reducing the displacement of small molecules, ions, and other diffusible substances. It also allows higher electron beam intensities to be used without degrading the sample [61,70–72]. When using chemical fixation methods, the preservation of the structure is compromised, as saturated lipids and sugars are not preserved and the distribution of biomolecules is altered [70]. Within a high-vacuum TEM system, the electron beam penetrates the vitrified sample, allowing both 2D and 3D images to be acquired [63,72]. Cryo-TEM can visualize nanostructures between 20 and 100 nm, including EVs, allowing detailed observation of their bilayer organization and thus facilitating studies on their biogenesis and morphology. In addition to cryo-TEM, atomic force microscopy in liquid (liquid AFM) has also been recently reported to reveal various morphological features of EVs, including identification of multilobed, round, elongated-bulging, single-lobed flat, and flat EV structures [73].

## 4. Reports on morphology variants of EVs

From the first observations of EVs using cryo-TEM, the presence of bilayer vesicles distinct from spherical morphology became evident, opening a new field of study focused on them. Despite this, research groups addressing the topic and the International Society for Extracellular Vesicles have not yet reached an international consensus on the classification of EV morphologies [15]. Therefore, several groups have developed their own EV classifications based on their analysis and experience in observing images obtained, mostly using cryo-TEM. This technique is considered the best approach for this type of study as it allows observation of the near-native EV structure, yielding fine details about the shape and structure of the membrane (Table 2). However, there is no universal agreement on many aspects of methodology in EV research, including the best methodology for enrichment as protocols vary between laboratories [90].

**Table 2.** Morphological classification of EVs. Created using BioRender.com.

Morphology	Type	Origin	Description	Reference
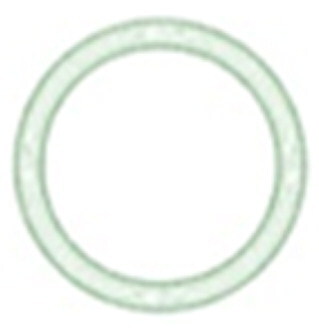	Single vesicle (Small or large light vesicles)	Human seminal fluid, TPH1, human plasma HMC-1, breast milk, urine, murine Sertoli cells, murine neuronal cells, Down syndrome mice model, follicular fluid, porcine ejaculate Alexandrium minutum* Dictyostelium discoideum* Sorghum bicolor* Arabidopsis thaliana* grapefruit juice *	Spherical vesicle with well-defined lipid bilayer	[74–79, 80–86*; 87*; 88*; 89*; 91]
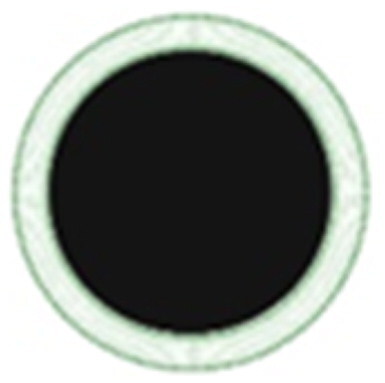	Electrodense (Small or large dark vesicles)	Human seminal fluid, TPH1, human plasma, HMC-1, breast milk, urine, murine Sertoli cells, porcine ejaculate Alexandrium minutum* Sorghum bicolor*	Characteristics of a single vesicle but with highly electrodense content (proteins, metabolites, lipids, and nucleic acids)	[75–79, 83–86*; 89*; 91]
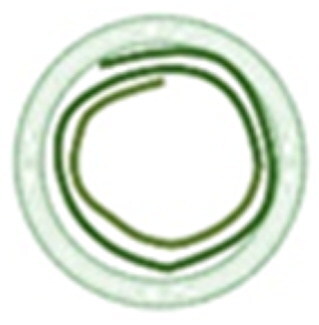	Lamellar (Myelinosomes)	Human seminal fluid, murine Sertoli cells, follicular fluid	From their membrane, they form lamellar layers	[77,75,86,82]
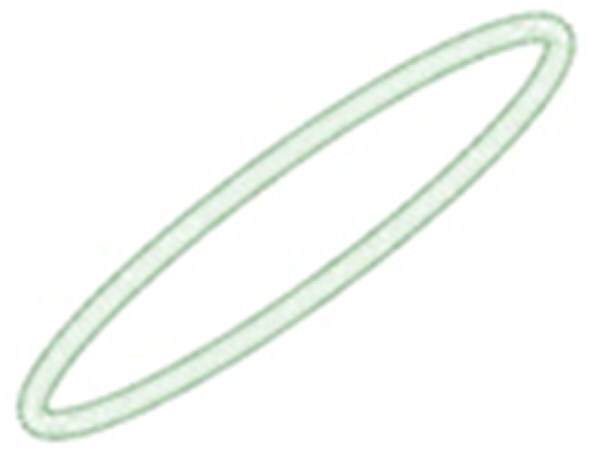	Tubular (Small or large)	Human seminal fluid, human plasma, HMC-1, breast milk, follicular fluid	Vesicles that are elongated in shape but retain a lipid bilayer	[74,75,77–79, 82, 91]
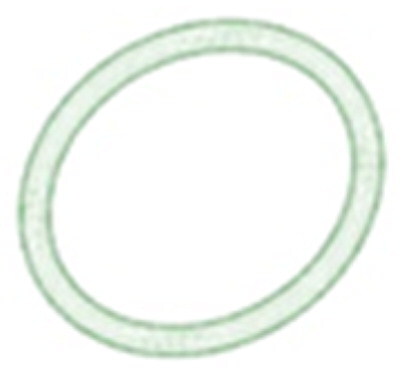	Oval	Human seminal fluid, HMC-1, follicular fluid grapefruit juice *	Single vesicle with its characteristic lipid bilayer but oval in shape	[75,79,82,88*]
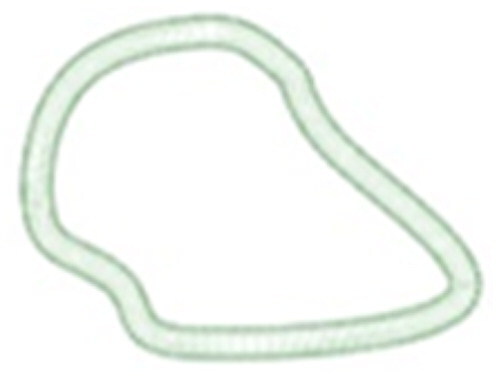	Pleomorphic (Low electron density)	TPH1, human seminal fluid, HMC-1, murine Sertoli cells, Down syndrome mice model, follicular fluid Dictyostelium discoideum*	This vesicle maintains its lipid bilayer but with irregular oval or spherical shapes, in some cases pear-shaped	[75,76,79,80,82,86,87*]
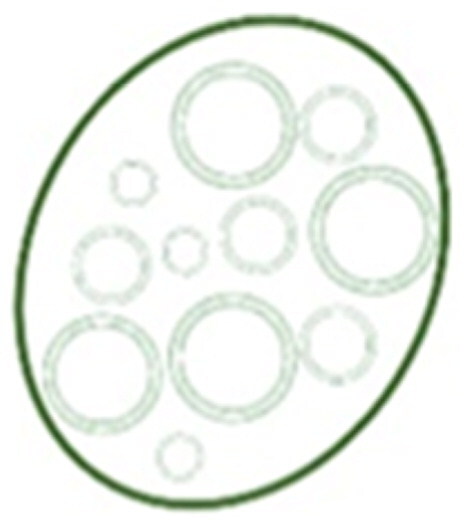	Vesicle sacs	Human plasma, human seminal fluid, follicular fluid	This structure is shaped like a sac without a lipid bilayer and contains more than 10 simple vesicles	[76,75,82]
Morphology subcategories	Type	Origin	Description	Reference
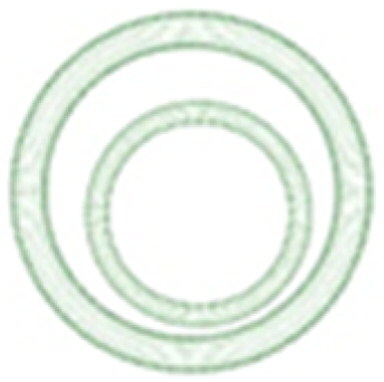	Double vesicle (Small or large)	Human seminal fluid, HMC-1, urine, Down syndrome mice model, porcine ejaculate grapefruit juice * Alexandrium minutum*	Single vesicle containing a smaller vesicle	[75,77,79,80,83,85,88*,^84^*]
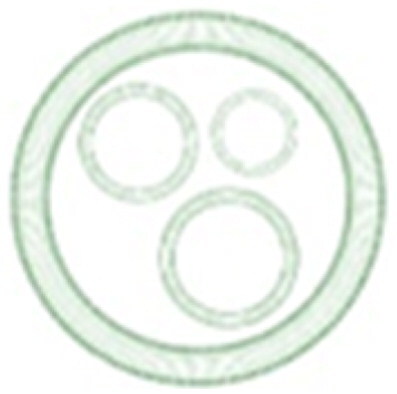	Multilayer	TPH1, human plasma, human seminal fluid, HMC-1, urine, murine Sertoli cells, murine neuronal cells, Down syndrome mice model, follicular fluid Dictyostelium discoideum* Sorghum bicolor* Arabidopsis thaliana*	A single vesicle contains more than two smaller single vesicles	[75,76,78,80–82,85–87*, 89*]
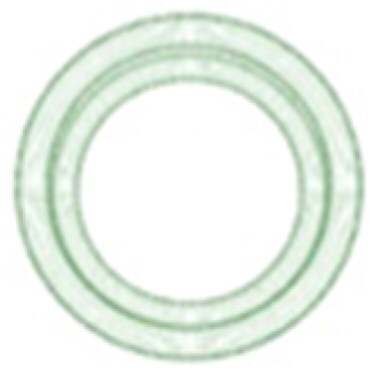	Double membrane	Human seminal fluid, Down syndrome mice model	Vesicle containing a double lipid bilayer	[75,80]
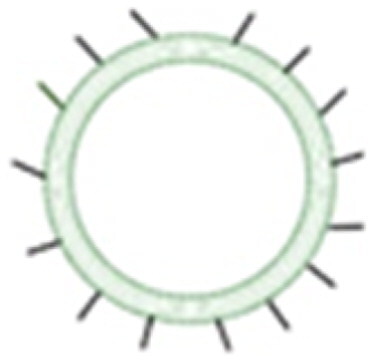	Coated membranes	Human seminal fluid, HMC-1	Vesicles with surface protuberances, electrodense spines protruding from the membrane or part of the membrane	[75,79]
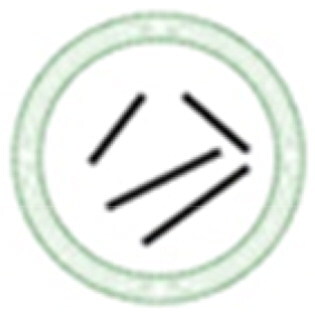	Filaments vesicles	HMC-1	Single vesicles containing parallel or randomly arranged filaments	[79]
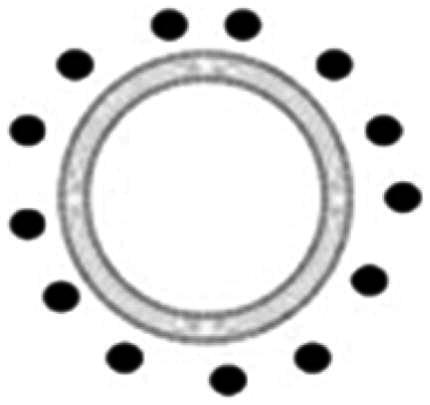	Small spherical structures around the vesicles	Human seminal fluid, THP-1, urine, porcine ejaculate	Small spherical structures (glycoproteins, lipoproteins, clathrin) are found around a single vesicle.	[77,76,85,83]
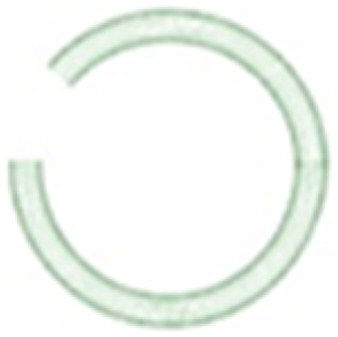	Broken membrane (incomplete)	Human seminal fluid, HMC-1 Dictyostelium discoideum*	Vesicles showing a rupture of the membrane	[75,79,87*]

The first evidence of variations in the morphology of EVs came from the observation of vesicles from human seminal fluid isolated by ultracentrifugation and sucrose gradient centrifugation and analyzed by cryo-TEM. According to the authors’ observations, in addition to the typical simple spherical vesicles with a well-defined lipid bilayer, they detected double-vesicle vesicles, which they described as smaller vesicles within a single larger vesicle, electrodense vesicles with characteristics of a single vesicle but with a highly electrodense content, and multilayer, elongated, or tubular. In some cases, the images obtained showed small spherical structures around the vesicles, which they described as possibly lipoproteins [77]. In 2013, using ultracentrifugation and cryo-TEM, morphological variants of EVs were identified in THP-1 mononuclear cell line cultures, including single, multilayer, and pleomorphic EVs surrounded by particles (which the authors suggested could be glycoproteins or lipoproteins). Interestingly, analysis of these EVs after freezing and thawing, as well as multiple rounds of ultracentrifugation, showed that these processes did not alter their morphologies or proportions. Only filtration and dialysis were observed to reduce the proportion of multilayer EVs [76]. In that same year, a study of EVs isolated from plasma of healthy adult donors using cryo-TEM coupled to tomography (cryo-ET) showed, in addition to simple typical EVs, the presence of single EVs with highly electrodense content, elongated or tubular EVs, and multilayer EVs originating from a larger vesicle containing three to six internal vesicles. The use of cryo-et al.lowed for the observation of the multilayer EVs within the larger vesicle and their individuality [78]. The following year, another study of EVs also from human plasma using cryo-TEM showed similar results, but added the morphology of EVs in vesicular sacs, structures containing more than ten vesicles, without a defined main vesicle. However, in this study, it was not entirely clear how the EVs were isolated [74].

Although there were increasing reports demonstrating the existence of morphological variants of EVs, it remained unclear whether the mechanical effects of centrifugal force during purification could be leading to the formation of multilayer EVs and their variants. In this regard, a 2015 study, limited to observing EVs in human ejaculate without isolating them and using cryo-ET, demonstrated the existence and suggested the natural generation, of multiple morphological variants of EVs, becoming a benchmark for variations in the shape of these structures. In human ejaculate, the following types of EVs were identified: single, oval, double, multilayer, lamellar, and incomplete vesicles (ruptured membrane), with small tubular, large tubular, pleomorphic and vesicular sacs shapes, and additionally, three subcategories: coated membranes, electrodense membranes, and double membranes (Table 2). Each morphology was quantified, with single vesicles representing 50% of all EVs found. These data strongly support the idea that ultracentrifugation and filtration are not responsible for the formation of multilayer, incomplete, or broken EVs seen in other studies, but rather that these variations are normally generated from the cell during vesicular biogenesis [75]. Similar results to human ejaculate were obtained when EVs were isolated by sequential centrifugation, ultrafiltration, and size exclusion chromatography from porcine ejaculate. In particular, these EVs were divided into small and large EVs, electrodense (rich in biomolecules), complex (double vesicles), and vesicles with small spherical structures around them, representing possibly lipoproteins, lipids, or nucleic acids [83].

More recent studies isolating EVs by ultracentrifugation from other human secretions, including breast milk (very rich in EVs), seminal fluid, follicular fluid, and urine, have also demonstrated the presence of EVs in a wide variety of forms. In breast milk, single, electrodense, and elongated (tubular) EVs were identified, the latter being confirmed as EVs by staining with annexin V coupled to gold particles to identify phosphatidylserine [91]. In the case of EVs from human seminal fluid, comparison with EVs secreted by Sertoli cells from mice showed no marked differences between the two samples revealing, in addition to the aforementioned EVs, dark EVs (spherical vesicles with a lipid bilayer and high electron density material inside), myelinosomes (defined as EVs with high electron density that are spherical in shape but whose membrane shows lamellar shapes), and pleomorphic EVs (irregular oval or spherical shapes) [86]. In follicular fluid from patients undergoing fertilization processes by intracytoplasmic sperm injection and with normal ovulatory cycles, authors reported a high variety of EVs, including single (which accounted for the highest percentage of EVs), oval, double, multilayer, large and small tubular, pleomorphic, vesicular sacs, and lamellar bodies [82]. In the urine of healthy patients, single, larger than 200 nm, electrodense, multilayer, clathrin-coated, and double EVs were found [85].

Morphological variation has also been documented when studying EVs from some cell lines by cryo-TEM. Thus, in EVs from human mast cells HMC-1, nine categories were identified: single, double, multilayer, small double, oval, small and large tubular, incomplete, and pleomorphic EVs, and three subcategories: coated, filamentous, and electrodense vesicles [79]. This classification is similar to that observed by Höög and Lötvall in human ejaculate, as mentioned above [75]. Another example came from a study analyzing EVs from murine neuronal cells, where single and multilayer EVs with a lipid bilayer were identified. Notably, when analyzing the multilayer EVs by cryo-ET, it was possible to confirm that the small vesicles were indeed inside the larger one and they were not artifacts due to superimposition [81]. In 2021, D’Acunzo et al. described the trend of subcategories starting from a single vesicle by analyzing EVs isolated from the hemiprosencephalon of a mice model of Down syndrome. They described triple-membrane (multilayer), pleomorphic, double, and single EVs with subcategories such as double membrane, double dense membrane, single membrane, and dense membrane. Interestingly, in each gradient of purification, the largest proportion still comprised single vesicles [80].

On the other hand, morphological variations of EVs are not exclusive to cells and fluids of mammals, as they have also been reported in EVs isolated by ultracentrifugation and analyzed by cryo-TEM from some microorganisms and plants. Thus, the dinoflagellate *Alexandrium minutum* produced double vesicles, electrodense irregular, electrodense rounded and rounded (single vesicles) EVs [84], while *Dictyostelium discoideum* produced small and large single, broken, multilayer, and pleomorphic EVs [87]. On the other hand, EVs from plants *Sorghum bicolor* and *Arabidopsis thaliana* showed at least three types of morphologies: single, multilayer, and electrodense EVs [89]. In the same order, EVs isolated from grapefruit juice showed single, double, and oval EVs [88]. Taken together, the evidence shows that morphological types are present as long as EVs are isolated and analyzed by cryo-TEM, regardless of their origin or isolation method used. Unfortunately, the molecular mechanism by which vesicle biogenesis gives rise to morphologically diverse EVs remains unknown.

As detailed in this review, there are different morphological classifications of EVs according to their origin and biological source. However, taken together, the EVs can be unified according to the reported evidence into seven main morphologies: single spherical vesicles, oval vesicles, electrodense vesicles (dark luminal content), lamellar vesicles (containing several layers of apparently rolled-up membrane), elongated or tubular vesicles, sac-like vesicles (large spherical or elongated vesicles containing several vesicles inside), and pleomorphic EVs. From these seven main morphologies, a series of subcategories (spherical in shape) have been described that increase the number of EV forms to at least 14 (Table 2).

Finally, one of the major current unknowns regarding EV morphologies is their origin. It remains unclear whether they can result from the various EV biogenesis pathways involving variable sets of proteins, lipids, and cellular conditions that can influence EV architectures. An even more intriguing question is whether specific functions are associated with each morphological type, a crucial point to resolve in this new branch of research, which would require the development of novel methodologies and tools for isolating the different variants.

## 5. Changes in the pattern of morphological variants of EVs are observed in non-infectious pathologies with cellular or metabolic dysfunction and in some infectious diseases

**Figure 2.**
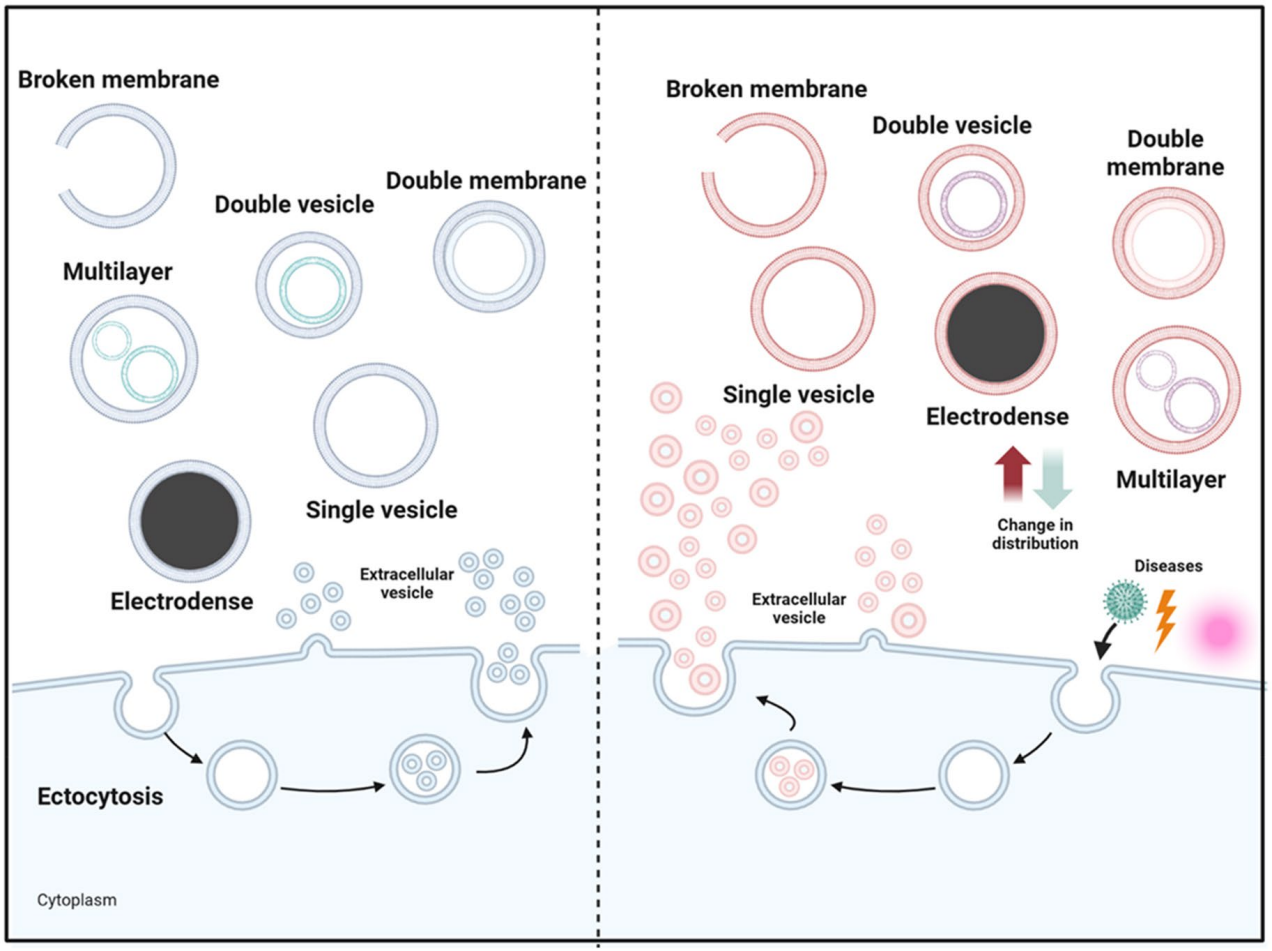
Alterations in EV morphology associated with disturbance of homeostasis. The left side depicts the regulation of EV morphologies under physiological conditions. In contrast, the right side shows that, in pathological states, including Gaucher disease, Parkinson’s disease, breast cancer, polycystic ovary syndrome, diminished ovarian reserve, obesity, type 2 diabetes, as well as infections by pathogens such as M1000 prion and Zika virus, EV biogenesis is altered. These modifications are reflected in changes in both the abundance and morphological diversity of EVs. Such alterations do not follow a uniform pattern but depend on the cellular context, resulting in a variable predominance of specific morphological subtypes. Created using BioRender.com.

During the 1980s and 1990s, several articles reported the quantification of EVs, demonstrating altered EV numbers in disease. Key reports included papers on elevated microparticles in transient brain ischemia and other infarctions [92] and also in diseases such as angina [93]  and Crohn’s disease [94]. It is now well accepted that in pathological conditions, EVs from aberrant cells are often secreted in large quantities into bodily fluids and possess properties that reflect specific disease states [95]. However, studies focused on changes related to morphological variations of EVs have only recently begun with the use of cryo-TEM. The process by which the various EV variants are generated and whether there is an association with changes in biological function remains a mystery. However, it is worth noting that during various non-infectious and infectious pathologies, the secreted EVs show alterations in the distribution pattern of the morphological types previously described (Figure 2).

In the case of non-infectious diseases, observations have been made in diseases that can be grouped so far as cellular or metabolic dysfunction. Such is the case of Gaucher′s disease, a recessive inherited lysosomal storage disorder triggered by mutations in the GCase gene, which encodes β-glucocerebrosidase. The main characteristic of this disease is GCase deficiency and the accumulation of glucosylceramide, which, in some patients, can result in the development of Parkinson’s disease [96]. EVs isolated by ultracentrifugation from plasma and whole blood of Gaucher′s disease patients and analyzed by cryo-TEM showed multilayer and electrodense vesicles, the latter lacking a lipid bilayer, a morphology that contrast with EVs from healthy donors, which showed typical single EVs. In particular, the electrodense EVs were 25 nm in size, suggesting to the authors that they are lipoproteins based on their observed size [96]. This work proposed that the variation in EV morphology was related to the lipid alteration characteristic of Gaucher′s disease, since the ESCRT-independent biogenesis of lipid-rich EVs is mediated by ceramides.

Parkinson’s disease, which, as mentioned, can occur as a consequence of Gaucher′s disease, is another condition in which morphological variants of EVs have been demonstrated. Parkinson’s disease is a progressive neurodegenerative disorder of the central nervous system affecting patients’ movement, resulting in symptoms such as tremors, stiffness, slowness of movement, and balance problems [97]. EVs isolated by ultracentrifugation from the cerebrospinal fluid of patients with this disease and observed by cryo-TEM showed five morphological types: single, double vesicle, double membrane, multilayer, and electrodense EVs. Comparison with EVs from patients with unrelated neurosurgical conditions, such as epidermoid cysts, vasoneural disorders, post-hypoxic encephalopathy, subarachnoid hemorrhage, or arachnoid cysts, showed that Parkinson’s patients had a 50% reduction in single EVs and up to a fourfold increase in the proportion of multilayer EVs [97]. Since Parkinson’s disease, like Gaucher’s disease, is strongly associated with alterations in metabolism and lipid composition in both the brain and peripheral tissues, it is likely that these alterations may contribute to the variations observed in EV morphology in these patients. However, this has not been proven and is the subject of intense study. Interestingly, alpha-synuclein has been reported to aggregate and be transported by EVs, which depends on the lipid composition of these structures. By influencing lipid distribution, it is theorized that cellular factors involved in EV transport during various cellular events affect the size and morphology of EVs [98–100]. This breakthrough was addressed to analyze the EVs of Parkinson’s patients by cryo-TEM and to determine the morphological changes in these vesicles [97].

As part of the characterization of EVs from plasma and whole blood of patients with breast cancer, EVs isolated by ultrafiltration and ultracentrifugation were analyzed. Interestingly, they also showed the same five morphological variants described in the Parkinson′s disease: single, double, multilayer, double-membrane, and electrodense EVs. In addition to the variants, an increase in double membrane EVs was observed in the plasma samples from breast cancer patients compared to healthy donors. Conversely, when analyzing EVs from patient′s whole blood, a decrease in electrodense, double membrane, and double vesicle EVs, concomitantly with a doubling in the proportion of single vesicles, was observed when compared to healthy donors. Additionally, an increase in the expression of miR-92a and miR-25-3p in the EVs of patients, which are related to increased angiogenesis and cell migration, was also found. Therefore, the authors proposed that this morphological phenomenon of EVs could play an important role in the tumor microenvironment [101].

Furthermore, in a study conducted in infertile women, vesicles were isolated from follicular fluid by ultracentrifugation and analyzed by cryo-TEM. The data obtained showed nine morphological variants consistent with those reported by (78]: single, oval, double, multilayer, incomplete, tubular, pleomorphic, saccular, and lamellar EVs. Interestingly, significant differences were observed between EVs from infertile patients with polycystic ovary syndrome (PCOS) and patients with diminished ovarian reserve (DOR), both undergoing *in vitro* fertilization. Compared to EVs from healthy donors, which show the same morphologies in different proportions, single vesicles decreased and oval and double vesicles increased in PCOS and DOR, while tubular vesicles increased only in PCOS and saccular vesicles only in DOR. Lamellar vesicles were present in a small proportion only in DOR. At the same time, overexpression of miR-210HG and miR-182 was found in the EVs of patients with DOR, while miR-25 showed underexpression. The evidence presented by the authors is the first to correlate the morphologies of EVs and miRNA content in this type of patient with infertility problems who have PCOS and DOR [102].

Cryo-TEM analysis of EVs in metabolic contexts, such as obesity and type 2 diabetes, has also revealed changes in their morphological structures. EVs were isolated by ultracentrifugation from visceral adipose tissue (VAT) and subcutaneous adipose tissue (SAT) cultures from obese patients with or without type 2 diabetes and healthy donors. In obese patients with or without type 2 diabetes, in addition to the single, double, multilayer, and double-membrane EVs, which are also observed in healthy donors, ruptured membrane and granular EVs were found. Interestingly, when comparing the four morphologies common to all groups, the proportion of double-membrane EVs was quadrupled in SAT samples from healthy individuals, while conversely, their proportion was halved in VAT samples from patients with obesity or type 2 diabetes. At the same time, the proportion of multilayer EVs increased four to five folds compared to controls. Finally, when comparing the EVs of SAT and VAT, regardless of the group, a notable increase in double vesicles was observed in VAT-derived EVs. The study authors suggest that these findings could be related to the lipid alterations characteristic of obesity, considering that high lipid levels affect EV biogenesis through the ceramide-mediated ESCRT-independent pathway, which could play an important role in their morphological modulation [103]. This evidence may be related to AMPK regulation, as it has been reported that reduced AMPK is associated with obesity, insulin resistance, and increased EV release [104]. Taken together, these findings show that there are significant changes in EV morphology during the aforementioned diseases, reflecting possible alterations in their biogenesis due to changes in the lipid profile or other components and factors involved in vesicle remodeling.

On the other hand, few studies have conducted in the context of infectious diseases, with only prion and Zika virus infections been reported to impact their structure and morphology. Thus, infection of murine GT1-7 neuronal cells with the human prion M1000 resulted in the secretion of EVs, isolated by ultracentrifugation and Optiprep gradient centrifugation and analyzed by cryo-ET, that exhibited single, double (double vesicles), and triple-membrane (multilayer) morphologies, similar to those observed in uninfected cells. However, as mentioned for some non-infectious diseases, variations were observed in the proportion of the vesicle types in the infected cells, with a significant increase in single-membrane and decrease in triple-membrane EVs, while double-membrane EVs remained unchanged [105]. Reports on the modulation of prion infections and their association with EVs have shown that in a prion-infected mouse model, isolated EVs had the ability to transport the prion protein isoform PrPSc associated with scrapie, which gives prion disease its infectious characteristics [106].

Similarly, in the case of Zika virus, infection of primary cultures of human umbilical vein endothelial cells (HUVECs) with the PRVAB59 variant resulted in the release of six morphological variants of EVs (single, double, multilayer, electrodense, ruptured membrane, and double membrane), of which, multilayer, electrodense, and ruptured membrane EVs were observed only in infected cells, while single and double EVs increased significantly compared to uninfected HUVECs. The authors suggest that Zika virus modulates EV biogenesis to generate unique morphological types and to increase their size, possibly to transport viral factors or even whole viral particles [107]. It should be noted that several reports have found that EVs generated by Zika infection have the ability to transport cellular and viral factors (E protein, NS1 viral RNA) that facilitate viral pathogenesis [108–110].

The examples above highlight the need to study the mechanisms underlying changes in EV biogenesis during disease as well as developing highly accurate isolation methods for separating the different morphological types of EVs, and fine-tuning correlation studies both *in vitro* and *in vivo*, evaluating the effect of EVs on target cells of interest or in passive transfer assays in experimental models, respectively. This will open a new field of study aimed at determining the possible biological impact of morphological variants of these structures and their use for diagnosis, monitoring, and treatment.

## 6. Distinguishing native EV morphology from isolation-induced artifacts

Based on the aforementioned studies, it is now accepted that EVs constitute an intrinsically heterogeneous population in size, shape, and composition, derived from several biogenesis pathways (endosomal vs. plasma membrane). This heterogeneity includes spherical (predominant), discoidal or elongated, and multilamellar or tubular (less frequent) morphologies. Although cryo-TEM studies carried out on unprocessed samples strongly support the notion that EV diversity exists *in situ* and is not exclusively the product of experimental manipulation [75,82], it is also known that a number of EV morphologies reported in the literature do not represent native states but rather distortions induced during purification and processing. One of the most representative examples is the *‘*cup-shaped*’* morphology, frequently reported in early studies of EVs analyzed by negative-stained TEM. Currently, it is well known that this structure arises as a consequence of the dehydration process and the lipid bilayer collapse during sample preparation, rather than being an intrinsic characteristic of EVs. In contrast, cryo-TEM analyses have revealed that, under near-physiological conditions, EVs primarily display a well-defined lipid bilayer spherical morphology, thereby emphasizing the importance of employing native preservation techniques to prevent misinterpretations [111].

Furthermore, experimental factors such as buffer conditions and ionic strength can affect the stability of EVs, promoting aggregation, fusion, or apparent changes in size. Likewise, isolation methods, particularly ultracentrifugation, can induce mechanical deformations of EVs, as well as the co-precipitation of protein aggregates and lipoproteins that can be mistaken for these structures [112,113]. Since these non-vesicular structures can have sizes and densities similar to EVs, their presence represents an additional source of ambiguity. The use of other purification techniques, such as SEC (size-exclusion chromatography), which excludes the use of centrifugal force, has been described as helpful in obtaining EVs closer to their native state, as well as reducing contaminants [73]. SEC combined with cryo-TEM, which in addition to helping preserve morphological and structural integrity allows easy identification of the lipid bilayer, constitutes a powerful tool to consolidate observations of native morphological variants of EVs.

The body of evidence suggests that the morphological heterogeneity of EVs should not be considered solely as an experimental phenomenon. Conversely, this heterogeneity probably reflects variations in biogenesis pathways, lipid and protein composition, and the molecular contents of the vesicles. Specifically, it has been proposed that endosomal vesicles, whether formed through ESCRT-dependent or ESCRT-independent mechanisms, generally display more consistent morphologies. In contrast, those originating from the plasma membrane may demonstrate greater structural diversity, a consequence of cytoskeleton-mediated budding processes and alterations in membrane tension [29]. Furthermore, the composition of lipids, particularly the presence of cholesterol, ceramides, and phosphatidylserine, is known to affect the stiffness and shape of membranes, while the inclusion of proteins and large molecular complexes can create regions that influence vesicle structure ([13,23,24], Kallury and LeBleu 2020). Additionally, the physiological state of the cell of origin, such as conditions of stress, activation, or infection, could modify both the biogenesis and composition of EVs, indirectly impacting their morphology. This suggests that, under controlled conditions, certain structural variations could have functional relevance and be associated with specific intercellular communication processes [3,111].

Taken together, it is important to highlight the need to adopt a critical and standardized approach to the morphological analysis of EVs. The combination of techniques that preserve the native state, along with biochemical and functional characterization, is necessary to distinguish between genuine structural features and experimental artifacts. In this context, EV morphology should be considered not only a descriptive criterion but also a potentially informative property regarding their origin, composition, and biological function.

## 7. Regulation of EV morphology

Membrane curvature is a key factor shaping cells, organelles, and transport intermediates such as vesicles and tubules, and it plays an active role in regulating trafficking and cellular functions. The molecular mechanisms of membrane curvature rarely act alone, but instead cooperate in diverse ways. During EV biogenesis, variations in lipid composition and membrane asymmetry can generate or stabilize curvature, while crowding of membrane proteins and the distribution of transmembrane domains can locally bend membranes. Additionally, reversible insertion of hydrophobic motifs into the lipid bilayer can induce curvature by disturbing lipid packing. Beyond individual molecules, oligomerized protein domains can form nanoscopic scaffolds that stabilize curved membranes, and cytoskeletal elements and molecular motors can provide larger-scale mechanical forces that shape membranes. These combined molecular mechanisms not only determine vesicle morphology but also influence membrane-related processes such as membrane scission, fusion, protein concentration, and enzyme activation, highlighting that EV structure and function arise from coordinated interactions between proteins, lipids, and cytoskeletal forces rather than proteins alone [114,115].

**Figure 3.**
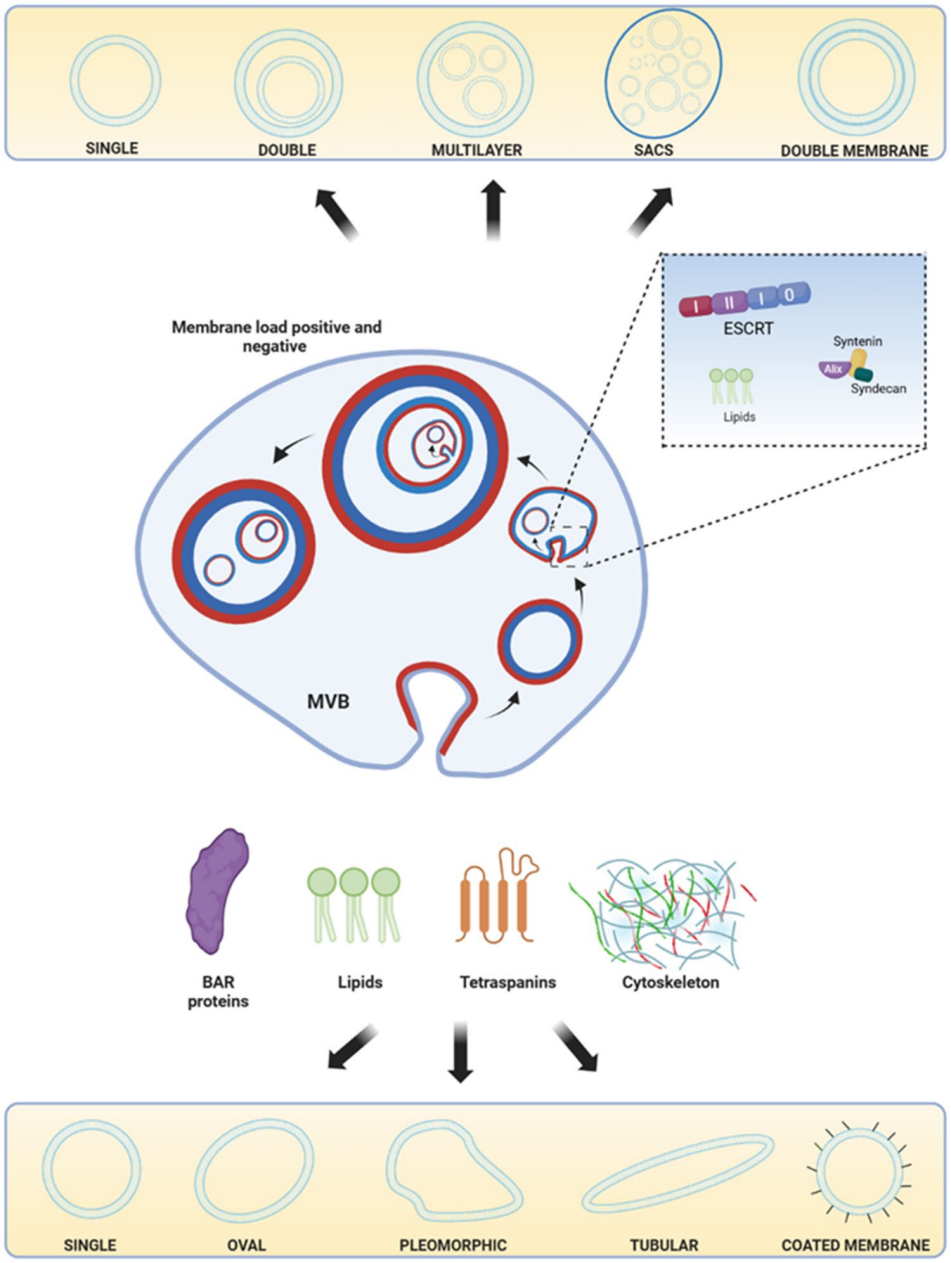
Regulation of EV morphology. The upper part of the image shows the possible regulatory pathways for the natural formation of variable ILVs in MVBs, originating from lipids and EV cargos such as the ESCRT complex, Alix, syntenin, and syndecan, resulting in single, double, multilayered, sacs, and double membrane vesicles. Sacs could also be complete MBVs released into the extracellular space. The lower part shows the regulation of membrane curvature by BAR proteins and lipids such as ceramide, tetraspanins, and cytoskeleton, giving rise to single, oval, pleomorphic, tubular, and coated membrane vesicles. Created using BioRender.com.

Regarding the pathways that regulate the expression of the EVs with different shapes, various hypotheses, not mutually exclusive, have been proposed. Among them is the involvement of proteins of the Bin-Amphiphysin-Rvs (BAR) superfamily, which are well known to regulate membrane curvature [116]. It has also been suggested that other proteins associated with membrane remodeling such as tetraspanins CD9, CD63, CD81, and CD82, which have a conical shape and are important in EV biogenesis and cargo sorting, would also play a relevant role in the generation of various EV morphologies [117–119]. Another hypothesis suggests the influence of lipids from the lipid bilayer itself such as ceramides, sphingomyelin, phosphatidylethanolamine, phosphatidylcholine, phosphatidylserine, and cholesterol, which are enriched in EV membranes and adopt conical and cylindrical shapes when they reassemble, particularly including the ESCRT-independent ceramide pathway [120]. Finally, cytoskeletal proteins associated with EVs, such as actin and tubulin, play important roles in EV formation, release, and function, both in their biogenesis and molecular cargo. These proteins can push or pull on membranes through polymerization or with the help of motor proteins, helping to define their morphology [23,24]. The key characteristics of these components and the corresponding levels of evidence on EV morphology are summarized in the Table 3. In Figure 3, it is shown possible pathways of biogenesis for shape and structure variants of EVs.

**Table 3.** Molecular regulators involved morphology and membrane curvature of EVs.

Molecule / system	Main effect on membrane	Predicted morphology	Level of evidence
BAR domain proteins (N-BAR, F-BAR, I-BAR)	Bind membranes and generate or stabilize curvature through protein scaffolding and insertion of amphipathic helices	Tubules and invaginations (/F-BAR); membrane protrusions (I-BAR)	*In vitro* experiments and multiscale computer simulations
Ceramides	Cone-shaped lipids that induce negative curvature and promote lipid domain reorganization	Endosomal invagination and intraluminal vesicle formation	High (cellular and biochemical evidence, especially in exosome biogenesis)
Tetraspanins (CD9, CD63, CD81, CD82)	Cone-shaped lipids, microdomains and cluster proteins, indirectly modulating curvature	Organized microdomains; budding of small vesicles	Monoclonal antibodies. physical mechanism still under study)
Actin cytoskeleton	Generates mechanical forces through polymerization and cortex remodeling	Membrane protrusions, budding, and vesicle neck constriction	High (microscopy, STED/Cryo ET)
ESCRT machinery (especially ESCRT-III)	Polymerizes to constrict and sever membranes from the cytosolic side	Inward budding and scission of intraluminal vesicles	Cryo-ET. *In vitro* studies.
Cholesterol	Modulates membrane order and rigidity; promotes lipid raft formation	Curved lipid domains and budding platforms	High. (biophysical and cellular studies)
Phospholipids	Lipid bilayers allow stability, fluidity and selective permeability	Planar membranes or closed vesicles (liposomes)	High/ well stablished.
Flippases / Scramblases	Generate lipid asymmetry between membrane leaflets	Spontaneous curvature due to leaflet area imbalance	Mass spectrometry. Fluorescence assays

### 7.1. Role of BAR proteins

BAR domain proteins are a superfamily of molecules essential for remodeling cellular membranes during processes such as endocytosis and synaptic vesicle recycling. They are characterized by their crescent-shaped dimeric structure, capable of binding lipid bilayers and inducing membrane curvature to various degrees, generating either positive or negative curvature [121] describe how they act as *‘*membrane sculptors*’*, transforming flat membrane surfaces into tubules or invaginations. Structurally, BAR domains form antiparallel dimers with a positively charged surface that electrostatically interacts with acidic phospholipids, allowing them to generate curvature by acting as scaffolds or by inserting amphipathic helices into the membrane [122] found that amphiphysin BAR domains preferentially bind to curved membranes, revealing their roles as both curvature sensors and generators. Furthermore, the BAR superfamily is diversified into subclasses (classical BAR, F-BAR, and I-BAR), each producing distinct curvature orientations. F-BAR domains generate positive membrane curvature leading to inward invaginations, while I-BAR domains induce negative curvature, promoting outward protrusions such as filopodia [123]. BAR proteins also play a crucial role in cytoskeletal dynamics by linking curvature formation with actin polymerization, allowing the mechanical forces from the cytoskeleton to enhance membrane bending during cellular remodeling [124]. Functionally, classical BAR proteins like amphiphysin and endophilin are essential for efficient synaptic vesicle endocytosis, enabling rapid recycling of vesicles post-neurotransmitter release. Disruption of endophilin has been shown to impair clathrin-mediated endocytosis at synapses, which negatively affects neural communication [125]. Importantly, mutations or dysregulation of BAR-domain proteins have been associated with human diseases, such as BIN1 (amphiphysin II), which is linked to centronuclear myopathy and Alzheimer’s disease. Research indicates that defective BIN1 disrupts T-tubule biogenesis in muscle cells, while genome-wide studies suggest that specific neuronal BIN1 isoforms may contribute to tau pathology, highlighting how defects in membrane sculpting can lead to widespread consequences for health [126].

There are a number of studies that demonstrate that BAR/I-BAR proteins are involved in the biogenesis mechanisms of EVs released from the plasma membrane (microvesicles) and in the formation of endocytic vesicles. In 2021, the proteomic analysis of the outer membrane vesicles (OMVs) of the bacterium *Shewanella oneidensis*, a potent metal ion reducer, revealed the identification of the BdpA protein with BAR-like activity. This protein was associated with the bacterium’s ability to produce redox-active membrane vesicles and outer membrane extensions (OMEs) with consistent diameter and curvature. A strain lacking BdpA produced lobed and disordered OMEs, while overexpression induced OME formation during the bacterium′s planktonic growth, a phase where they do not usually form, all of which analyzed by cryo-TEM [127]. In 2022, HEK293T cells transfected with the IRSp53 protein, belonging to the I-BAR protein family, were used to demonstrate the participation of this protein in EV budding, a process that requires Arp2/3 activity, a complex that regulates the initiation of actin polymerization, but it was independent of TSG101, a key protein of the ESCRT-I complex considered an exosome marker [128]. More recently, it was reported that the expression of the I-BAR-F3 variant in CHO cells increases the production of EVs, which, in turn, are taken up by neighboring cells [129]. Moreover, another recent study details how EVs containing BAR proteins (MIM/IRSp53) derived from cell protrusions, rather than EVs containing tetraspanin CD63 derived from endosomes, can mediate functional protein transfer (Rac1 and Cas12f) between cells with the same efficiency as a microinjection, proposing protrusion-derived EVs by MIM as the best potential for the transfer of bioactive molecules between cells [130]. The above observations suggest that BAR proteins play a relevant role in the biogenesis and composition of certain populations of EVs, especially those derived from protrusions, and that they could therefore be involved in the morphological variations observed in certain diseases.

### 7.2. Role of lipids

In biological membranes, lipids do not only provide structural support but also actively influence membrane remodeling processes such as vesicle formation and membrane fusion. Lipids play a fundamental role in EVs, as they constitute the structural basis of these vesicles [120]. Among them, the sphingolipid ceramide is considered a key lipid in EV biogenesis. Ceramide can accumulate in specific regions of the plasma membrane, forming ceramide-rich platforms, which promote negative curvature necessary for protein incorporation and EV budding from MVBs [131]. These domains promote structural rearrangements by clustering membrane components and altering membrane curvature, thereby facilitating processes such as vesicle budding, fusion, and intracellular trafficking. High-resolution imaging studies have shown that a significant fraction of membrane ceramides is concentrated in nanoscale platforms composed of multiple ceramide molecules. Moreover, when ceramide levels increase, both the number and size of these platforms grow, suggesting that ceramide enrichment enhances membrane reorganization and curvature changes associated with vesicle dynamics [132]. On the other hand, phosphatidylethanolamine, composed of two fatty acid acyl chains esterified to a glycerol molecule, exhibits conical geometry, which also promotes negative curvature of the cytoplasmic membrane, both in the cell and in EVs [133]. Phosphatidylserine (PS), for its part, is an anionic phospholipid predominantly restricted to the inner leaflet of the plasma membrane through the action of flippases. During apoptosis, PS becomes externalized as an *‘*eat-me*’* signal. Recent studies highlight how defects in scramblase or flippase activity disturb PS asymmetry, contributing to immune dysfunction and coagulation disorders [134,135]. The negatively charged headgroup of PS, in addition to recruiting signaling proteins containing PS-binding domains, experiences electrostatic repulsion which, when exerted asymmetrically, is predicted to favor membrane curvature [136].

Regarding sphingolipids, their long, saturated acyl chains interact tightly to form ordered microdomains, often cholesterol-rich regions known as lipid rafts, where signaling receptors cluster. Recent biophysical studies demonstrate that sphingolipid packing strongly influences membrane rigidity and protein organization, modulating downstream signaling pathways related to apoptosis and inflammation [137,138]. Cholesterol, instead, is a rigid, planar sterol that inserts between phospholipid tails and fine-tunes membrane fluidity, permeability, and thickness. Its steroid ring system allows it to stabilize ordered phases while preventing excessive membrane stiffness, serving as a dynamic structural regulator. Advances in cryo-EM and molecular dynamics reveal that cholesterol interacts cooperatively with sphingolipids and with specific membrane proteins to regulate nanoscale clustering essential for signal transduction and receptor activation [139]. Although cholesterol is not a classic *‘*bend-inducing*’* lipid like ceramides, its role as a key modulator of the mechanical behavior of the bilayer and of the formation of lipid domains that indirectly influence the curvature, stiffness, and organization of the extracellular vesicle (EV) membrane is recognized [119,140].

Disruptions in lipid metabolism are now recognized as major drivers of disease. Alteration in sphingolipid pathways have been linked to neurodegenerative conditions such as Alzheimer’s disease, Parkinson’s disease, and multiple sclerosis, where imbalances in ceramide and sphingosine-1-phosphate impair neuronal signaling and membrane stability [141]. Cholesterol dysregulation plays critical roles in metabolic syndrome, atherosclerosis, and hepatic steatosis, as well as in neurodegeneration due to defective sterol homeostasis [142]. Moreover, defective PS regulation contributes to antiphospholipid syndrome and thrombosis by altering activation of coagulation factors, and viruses such as SARS-CoV-2, Ebola, and Zika exploit PS-mediated *‘*apoptotic mimicry*’* to enter host cells. These findings underscore the crucial role of membrane lipids in both normal physiology and disease [143].

### 7.3. Role of tetraspanins

Understanding membrane proteins and their structural variants is crucial because their form dictates how cells communicate, organize their membranes, and respond to their environment. Even small changes in these proteins can disrupt cellular function and contribute to diseases such as cancer, neurodegeneration, metabolic disorders, and viral infections. Recognizing their roles helps identify biomarkers and develop targeted therapies that restore membrane balance. Recent studies have described a new perspective on the function of tetraspanins. These proteins are involved in the regulation of processes such as immunity, vision, renal function, fertilization, muscle regeneration, and various cellular dynamics, as well as in pathogen infection, cancer regulation, and the biogenesis and function of EVs [118]. Tetraspanins are small transmembrane proteins, approximately 200 to 300 amino acids in length, with varying degrees of glycosylation. Their main function is to act as membrane protein organizers. Structurally, this family of proteins is characterized by the presence of four transmembrane domains, which contribute to the formation of two extracellular loops: one small (EC1) and one large (EC2) [144]. The EC1 loop contains conserved Cys–Cys–Gly motifs, as well as other cysteine residues, while the EC2 loop promotes the formation of disulfide bridges and has a conserved structural domain in the form of a triple-helix, unique to certain tetraspanins, such as CD81, which are present in EVs [145,146].

It has been reported that CD81 can associate with cholesterol, as evidenced by bioinformatic simulations. This interaction allows the tetraspanin to adopt open or closed conformations, giving it significant structural flexibility within the cytoplasmic membrane [147]. Of particular interest is the description of how the structure of several tetraspanins, such as CD9, CD63, CD81, and CD82, acquire a conical conformation due to the arrangement of their four transmembrane domains, promoting their localization in regions of high curvature of the plasma membrane, such as tubular structures, microvilli, or highly curved domains [146,148–150]. This opens up a new perspective in which tetraspanins present in EVs, such as CD9, CD63, CD81, and CD82, possibly participate in the modulation and organization of the structure of EVs.

### 7.4. Role of the cytoskeleton

Extracellular vesicles are regulated by a coordinated interplay of proteins, lipids, and cytoskeletal components that collectively shape their morphology and biogenesis. Proteins containing BAR domains, known for their capacity to sense and induce membrane curvature, interact closely with specific lipids, such as ceramides. These lipids can modulate membrane curvature, thereby contributing to vesicle stability and facilitating their formation and release. Here, the cytoskeleton plays a crucial role by providing mechanical support and enabling the intracellular transport of these structures [151, Kong et al. 2023; [153] 151–156,^152^]. Structures such as microtubules and actin filaments facilitate the movement of lipid droplets within the cytoplasm, increasing the probability of contact and fusion. When these cytoskeletal components are disrupted, droplet mobility decreases, thereby limiting fusion and resulting in smaller lipid droplets. This suggests that the interaction between membrane lipids and the cytoskeleton is an important factor in vesicle-like dynamics, since cytoskeletal organization can influence membrane remodeling, droplet interactions, and ultimately lipid storage processes [157].

## 8. Conclusions and future prospects

The evidence described in this review shows that EVs are highly dynamic in structure, giving rise to diverse vesicular morphologies that manifest during the organism’s natural physiological state but whose pattern and proportions can vary under pathological conditions. Although most related studies have been conducted to date in mammals, publications are beginning to appear demonstrating that EV morphological variations are also present in microorganisms and plants, which will likely lead to the demonstration that this is a conserved biological process in nature.

Until just over a decade ago, EVs were thought to be simple spherical structures with a well-defined lipid bilayer, generally analyzed using electron microscopy techniques such as TEM and SEM. However, the use of cryo-TEM and its variants has opened a new field of study for EVs in terms of their morphology, revealing that they can also present as multivesicular, pleomorphic, or tubular structures, that their membrane can be double or lamellar, and that their contents can be electron-dense, among other prominent features observed by various research groups. The morphological variability of EVs suggests the existence of different subpopulations that can possess distinct functions and biochemical properties.

Several authors have pointed out that cryo-TEM allows EVs to be observed in an *‘*almost*’* native state [158,159]. By avoiding interactions with additional chemical agents or dehydration processes, this technique is considered one of the most suitable for evaluating the morphology of EVs in conditions as close as possible to their physiological state. Cryo-TEM technique, based on the instantaneous vitrification of the sample by freezing, has become fundamental to this field of study, as it allows visualization of EVs in their near-native form. Evidence suggests that TEM and SEM do not allow visualization of ultrastructural details of native EVs, mainly limiting themselves to identifying only the surface features of vesicles that may have been modified in their structure during sample processing. Consequently, EVs viewed by TEM or SEM appear to have only characteristic size and shape features that define it as single spherical vesicles. Pleomorphic and tubular morphologies are possibly missed in TEM and SEM tests because they do not meet the size and spherical shape that usually characterize EVs. In any case, some artifacts may arise from the air-water interface, ice thickness, vitrification process, and freeze-induced stress may occur, which can alter morphology and generate folds or cracks [160,161]. Likewise, the air-water interface can skew the orientation of macromolecules, favoring uneven distribution and possible damage to the molecule of interest [162]. However, these effects would not compromise the structures described in this review. In this context, cryo-TEM studies have been conducted on viral particles with icosahedral symmetry, such as MS2, RRV, and DENV2, with the aim of comparing symmetrical components that, in principle, should exhibit an identical structure and not be affected by sample preparation—including the use of NTE buffer and colloidal gold—or by exposure to the electron beam. Interestingly, it was observed that neither freezing nor electron irradiation induced contraction of the viral particles; damage was limited to regions located in areas close to the surface of the vitreous ice [163].

Considering all of the above, the observed EV variants are unlikely to be the result of an artifact caused by purification procedures as they have, in some cases, been described in unprocessed samples such as ejaculate [75]. However, it is important to exercise caution regarding this issue and to comply with the guidelines of the MISEV (Minimal Information for Studies of Extracellular Vesicles). The latest edition of the MISEV guide [15] points out that electron microscopy techniques such as TEM, SEM, and cryo-TEM are not necessarily interchangeable and do not guarantee comparable image quality. In particular, the desiccation conditions inherent to TEM and SEM can alter the morphology of EVs, generating artifactual structures with a *‘*cup*’* appearance that are not observed under hydrated conditions. Additionally, there are biases associated with image acquisition and field of view selection. Therefore, it is recommended that studies analyzing EVs using cryo-TEM detail the software version used, acquisition settings, sample preparation method, calibration procedures, and areas selected for analysis. In this regard, MISEV indicates that the different variants of electron microscopy are capable of detecting EVs regardless of their size; however, performance tends to be underestimated in the case of larger vesicles compared to smaller ones. For this reason, cryo-TEM offers clear visualization of the lipid bilayer and preserves a morphology close to the native state, as it avoids the dehydration processes characteristic of TEM and SEM. However, although it allows the surface of EVs of various sizes to be observed, the analysis of smaller vesicles remains technically challenging [159].

Interestingly, studies reveal that changes in the proportion or expression of these EV morphologies may be associated with high-impact public health pathologies such as metabolic and neurodegenerative diseases, cancer, reproductive system disorders, and prion and viral infections. Therefore, changes in morphology and proportions could indicate that EV structure is regulated by specific pathways that can be influenced by lipid metabolism, cellular stress, or interaction with pathogens. Currently, the major question is to identify the molecular mechanisms that determine the biogenesis of each EV morphological type as well as finding a way to isolate each morphological type to analyze its cargo in relation to its morphology and, even more challenging, whether changes in EV structure and cargo are a consequence or a cause of the diseases to which they have been linked, and what their biological function is. While there are proteins that remodel and give curvature to the membrane, such as those of the BAR family, lipids that form the structure of the lipid bilayer of the plasma membrane, and conical tetraspanins that are present in the structure of certain EVs such as exosomes, it is of utmost importance to determine whether these biomolecules, separately or together, play a leading role in modulating various morphological types.

Currently, there is no method that allows for the specific isolation of each morphological type of extracellular vesicle (EV). Therefore, future studies should focus on developing strategies that allow for the separation of different subpopulations, enabling *in vitro* and *in vivo* evaluation and thereby, allowing to determine whether they are biochemically and functionally distinct and whether they are involved in the development of associated diseases, or are merely a consequence thereof. In this sense, methodologies such as microfluidic chips with integrated immunoaffinity capture, acoustic-based nanofiltration, pressure or electrophoretic-driven nanoporous membrane filtration, nanoscale deterministic lateral displacement, electrokinetics, and others, are under development. This approach would contribute to broadening our understanding of the biological functions associated with each morphological type present in EVs. Finally, the identification of each type of EVs in terms of its structure is expected to be useful as a potential biomarker for the early diagnosis of certain pathologies as well as for evaluating active disease and therapeutic response, or to understand cell communication in a state of homeostasis. Therefore, unraveling the biological aspects of the morphological variants of EVs is emerging as a field of study with potential applications in biomedicine and biotechnology.

## Data Availability

Data sharing is not applicable to this article as no data were created or analysed in this research
